# Axial Identity of Spinal Cord Neural Progenitor Cell Grafts Is Dispensable for Regeneration and Functional Recovery After Spinal Cord Injury

**DOI:** 10.3390/cells15060497

**Published:** 2026-03-11

**Authors:** Ashley Smith, Valerie Dietz, Joseph D. Hoppe, Gillian Imrie, Grant Lee, Amy Leonards, Vipin Jagrit, Abigail Evans, Tucker Gillespie, Bryson Gottschall, Benard Inskeep, Prakruthi Amar Kumar, Logan Friedrich, Murray G. Blackmore, Isabella Farhy-Tselnicker, Jennifer N. Dulin

**Affiliations:** 1 Department of Biology, Texas A&M University, 301 Old Main Drive, Interdisciplinary Life Sciences Building (Room 3126A), College Station, TX 77843, USAvalerieanne1079@tamu.edu (V.D.); vjagrit.007@tamu.edu (V.J.); brysoncg@tamu.edu (B.G.); binskeep@tamu.edu (B.I.); prakruthi.amarkumar@ucsf.edu (P.A.K.); ifarhy@bio.tamu.edu (I.F.-T.); 2 Solon High School, Solon, OH 44139, USA; grantlee5168@gmail.com; 3 Department of Biomedical Sciences, Marquette University, Milwaukee, WI 53304, USA; 4 Texas A&M Institute for Neuroscience, Texas A&M University, College Station, TX 77843, USA

**Keywords:** neural stem cells, neural progenitor cells, spinal cord injury, cell transplantation, forelimb function, motor recovery, regional identity, anterior, posterior

## Abstract

Neural progenitor cell (NPC) transplantation is a promising strategy for spinal cord injury repair, as graft-derived neurons can integrate into host circuitry and promote functional recovery. While the brain-regional and dorsoventral identities of NPCs are known to influence graft composition and performance, the importance of axial (rostrocaudal) identity, specifically whether NPCs must be matched to the spinal level of injury, remains poorly understood. To address this, we compared outcomes following transplantation of NPCs isolated from the anterior embryonic spinal cord (A-NPCs) versus the posterior spinal cord (P-NPCs) in a mouse model of C5 cervical dorsal column injury. Following transplantation, NPCs retained their intrinsic molecular axial identities; P-NPC grafts maintained significantly higher expression of the lumbar-associated gene HoxC10 and possessed a higher proportion of Chx10-high V2a neurons compared to A-NPCs. Despite these maintained molecular differences, A-NPC and P-NPC grafts were indistinguishable in neuronal and glial density, axon outgrowth, and their ability to support host axon regeneration, including the corticospinal tract. Long-term behavioral testing and retrograde transsynaptic tracing revealed no significant differences between groups in the recovery of skilled pellet reaching, grip strength, or synaptic integration with host cervical motor circuitry. These findings demonstrate that although transplanted NPCs retain their molecular axial identity in the adult injured environment, this identity is not a primary determinant of anatomical integration or functional outcome. Our findings suggest a degree of plasticity in graft-host interactions and indicate that strict segment-matching is not essential for the efficacy of NPC-based therapies in spinal cord injury.

## 1. Introduction

Spinal cord injury (SCI) causes immediate and permanent loss of neurological function, in large part due to the failure of the central nervous system (CNS) to regenerate neuronal connections lost by injury. Over the last four decades, a large number of studies have demonstrated that transplantation of neural stem cells and neural progenitor cells (NPCs) derived from fetal rodent tissue or human pluripotent stem cells can successfully provide new neurons to the lesioned spinal cord. Graft-derived neurons have been shown to support the establishment of new graft/host neural connections, attenuate glial cell reactivity, and promote modest recovery of grasping and walking function [1,2,3,4,5,6,7,8,9,10,11,12,13,14,15,16,17,18]. However, our understanding of the mechanism(s) by which transplanted NPCs support improved recovery of motor function remains incomplete. As efforts to evaluate the safety of human NPC transplantation in clinical studies are underway [19], it is critical to utilize preclinical animal models to ask basic questions about graft/host interactions so that the efficacy of NPC grafts can be improved to promote greater functional recovery.

It has become increasingly apparent that the regional identity of grafted NPCs is an important consideration in grafting studies [20]. In models of traumatic brain injury and Parkinson’s disease, area-specific (homotopic) cells transplanted into brain lesions promote enhanced functional recovery compared to heterotopic cell grafts [21,22,23,24,25]. In SCI models, transplantation of NPCs caudalized to spinal cord identities have been shown to promote better outcomes than grafts with rostral (brain or brainstem) identities [26,27]. However, beyond spinalization, it is not yet clear whether spinal cord axial identity affects graft performance.

In the intact spinal cord, axial identity is a key factor determining function at distinct spinal levels. Motor circuits are highly specialized along the rostrocaudal axis, with neurons in the cervical, thoracic, and lumbar regions controlling forelimb, trunk, and hindlimb movements, respectively. This functional specialization arises from intrinsic neuronal properties, patterns of connectivity, and the interplay with local and descending inputs [28,29,30,31,32,33,34,35,36]. Given this organization, it is plausible that NPCs with spinal cord segment-specific identities may integrate more effectively into host circuitry, thereby optimizing functional recovery after SCI. However, the extent to which axial identity influences graft survival, differentiation, and circuit integration has not yet been studied. To address this knowledge gap, we designed a study to investigate how transplantation of NPCs derived from the anterior embryonic spinal cord (A-NPCs) versus from the posterior embryonic spinal cord (P-NPCs) affected outcomes following cervical spinal cord injury. By thoroughly testing and ultimately ruling out axial identity as an important determinant of graft efficacy, the results provide important guidance for the ongoing refinement of neural transplantation strategies for SCI.

## 2. Methods

### 2.1. Ethics Statement

Animal experiments were conducted in accordance with the *NIH Guidelines for Animal Care and Use of Laboratory Animals* and were reviewed and approved by the Texas A&M University Institutional Animal Care and Use Committee. Experimental design and surgical procedures were implemented with the goal of minimizing animal discomfort and reducing distress.

### 2.2. Animals

**Figure 1:**
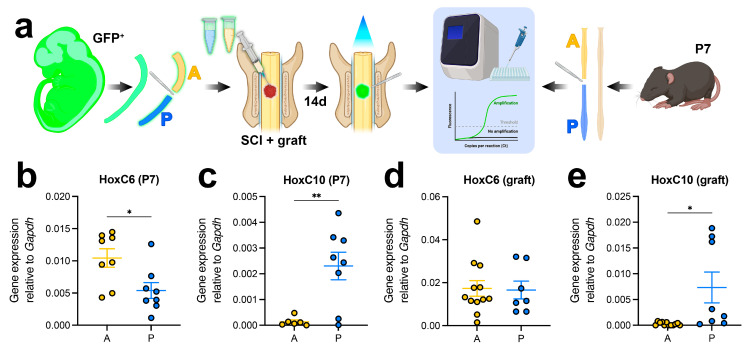
*Hox* gene expression in age-matched graft and postnatal spinal cord tissue. (**a**) Experimental schematic. Left: spinal cords were removed from GFP+ E12.5 embryos and anterior (A) and posterior (P) halves were separated. Neural progenitor cells were isolated from A and P spinal cord and transplanted into sites of cervical (C5) spinal cord injury. Two weeks later, GFP+ grafts were harvested and tissue was processed for RT-qPCR. Right: P7 pups were euthanized and their spinal cords were removed. Anterior and posterior regions of spinal cord were separated and RNA was isolated for RT-qPCR. (**b**) HoxC6 expression in P7 spinal cord. (**c**) HoxC10 expression in P7 spinal cord. (**d**) HoxC6 expression in grafts 14 days post-transplantation. (**e**) HoxC10 expression in grafts 14 days post-transplantation. All data are mean ± SEM. * *p* < 0.05, ** *p* < 0.01 by Welch’s *t* test. Created in BioRender. Dulin, J. (2026) https://BioRender.com/d3h2r2b (accessed on 6 March 2026).

**Figure 2:**
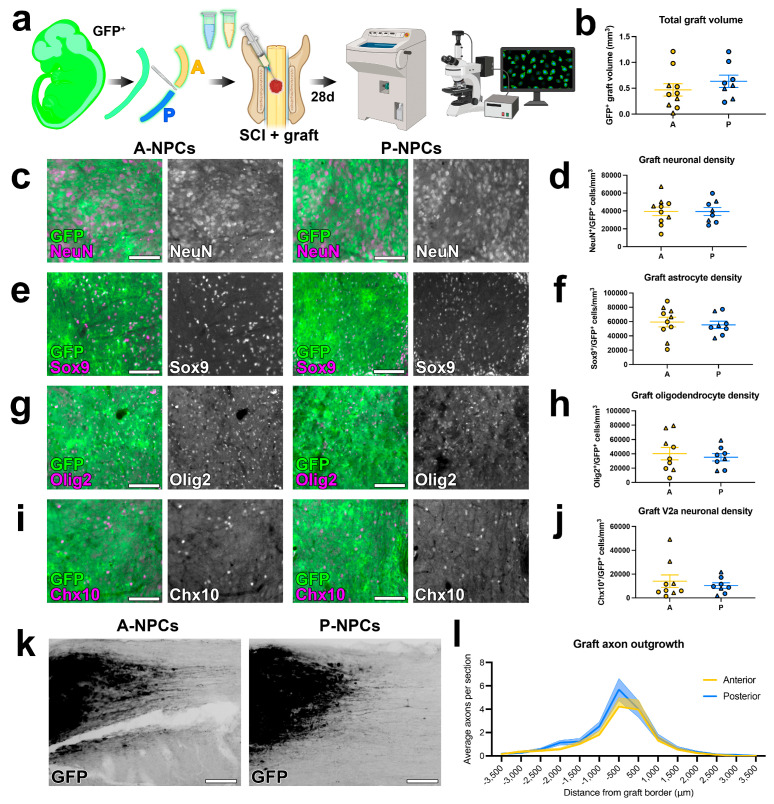
Graft axial identity does not affect graft cell composition or axon outgrowth. (**a**) Experimental schematic. GFP+ A-NPCs (anterior NPCs) or P-NPCs (posterior NPCs) were transplanted into sites of cervical (C5) SCI and allowed to mature for 28 days, then tissue was processed for immunohistochemistry. (**b**) Anterior/posterior identity does not impact graft volume. (**c**,**e**,**g**,**i**) Fluorescence images of (**c**) neurons, (**e**) astrocytes, (**g**) oligodendrocytes, and (**i**) V2a interneurons in grafts. (**d**,**f**,**h**,**j**) Quantification of cell density in grafts for each cell type. (**k**) Images of GFP+ axon outgrowth from grafts. (**l**) Quantification of axon outgrowth from grafts; negative distances indicate caudal from the graft and positive distances indicate rostral to the graft. All data are mean ± SEM. Triangular data points indicate female subjects and circular data points indicate male subjects. Scale bars = 100 μm. All data are mean ± SEM. No significant differences identified by Welch’s *t* test (**b**,**d**,**f**,**h**,**j**) or two-way ANOVA (**l**). Created in BioRender. Dulin, J. (2026) https://BioRender.com/mqfh9re (accessed on 6 March 2026).

**Figure 3:**
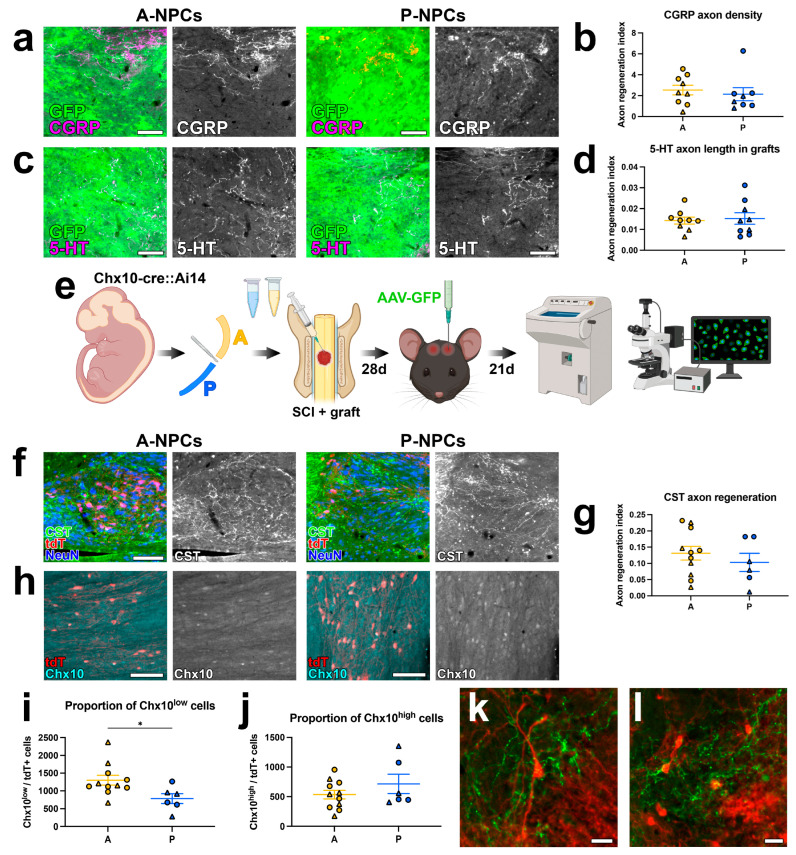
Graft axial identity does not affect host axon regeneration into grafts. Panels (**a**–**d**) show data from GFP+ grafts generated in experiments described in Figure 2a. (**a**) Fluorescence images of host CGRP+ axon regeneration into grafts. (**b**) Quantification of host CGRP+ axon regeneration into grafts. (**c**) Fluorescence images of host 5-HT+ axon regeneration into grafts. (**d**) Quantification of host 5-HT+ axon regeneration into grafts. (**e**) Experimental schematic corresponding to data in panels (**f**,**g**). Spinal cords were harvested from Chx10::Ai14 mouse embryos and dissected into A and P components, then grafted into sites of C5 SCI. Four weeks later, corticospinal tract (CST) axons were anterogradely traced with AAV-GFP. Tissue was collected 3 weeks post-AAV injections. (**f**) Fluorescence images of CST axon regeneration into grafts. (**g**) Quantification of CST axon regeneration into grafts. (**h**) Fluorescence images of Chx10 immunoreactivity in tdTomato+ V2a graft neurons. (**i**,**j**) Quantification of (**i**) Chx10-low and (**j**) Chx10-high immunoreactivity in tdTomato+ V2a graft neurons. (**k**,**l**) Fluorescence images of GFP+ CST axon terminals closely associated with graft V2a neurons. Triangular data points indicate female subjects and circular data points indicate male subjects. All data are mean ± SEM. * *p* < 0.05 by Welch’s *t* test. Scale bars = 100 μm (**a**,**c**,**f**,**h**), 20 μm (**k**,**l**). Created in BioRender. Dulin, J. (2026) https://BioRender.com/bb4deyz (accessed on 6 March 2026).

**Figure 4:**
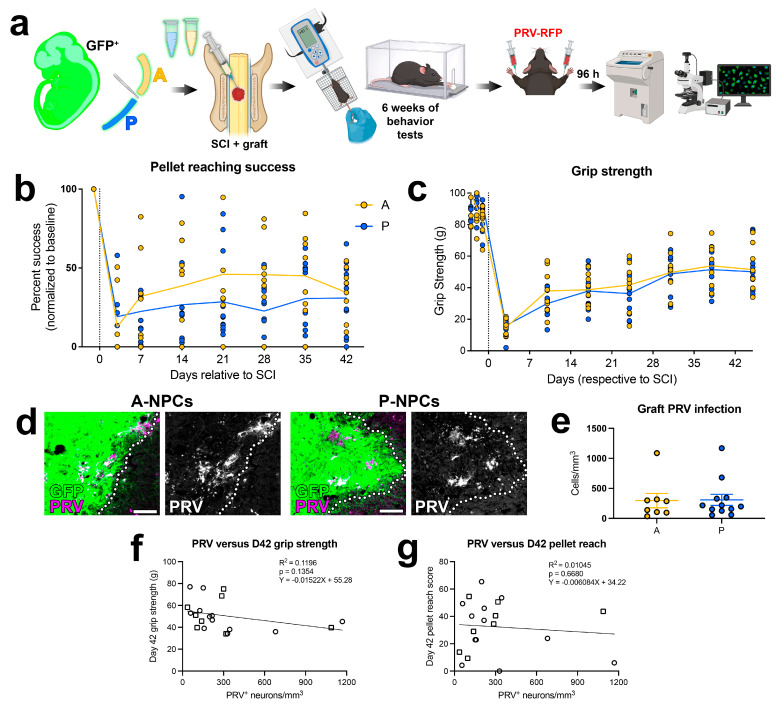
Graft axial identity does not influence recovery of forelimb motor function following SCI. (**a**) Experimental schematic. GFP+ A-NPCs or P-NPCs were transplanted into sites of C5 SCI in male mice. Forelimb behavioral assessments were performed weekly for 6 weeks beginning at 3 days post-SCI, then PRV-RFP was injected into bilateral forelimb nerves. Animals were sacrificed for histology 96 h later. (**b**) Graph of pellet reaching scores post-SCI (post-injury scores are normalized to baseline, pre-injury scores of individual subjects). (**c**) Graph of grip strength scores. (**d**) Fluorescence images of PRV+ neurons in GFP+ grafts (grafts outlined with dotted lines). Scale bars = 100 μm. (**e**) Quantification of PRV+ neuron density in grafts. All data in panel (**e**) are mean ± SEM. (**f**) Simple linear regression analysis of the density of PRV+ neurons in the graft vs. Day 42 post-SCI grip strength scores. (**g**) Simple linear regression analysis of the density of PRV+ neurons in the graft vs. Day 42 post-SCI pellet reaching scores. For panels (**f**,**g**), square data points indicate A-NPC subjects and circle data points indicate P-NPC subjects. All data are mean ± SEM. No significance differences were found by two-way repeated measures ANOVA (**b**,**c**) or Welch’s *t* test (**e**). Simple linear regression analysis was used in (**f**,**g**). Created in BioRender. Dulin, J. (2026) https://BioRender.com/ji52p1q (accessed on 6 March 2026) and ChatGPT 5.2.

A total of 172 mice were used for this study, including 122 10–14 week-old C57BL/6J mice (#000664, Jackson Laboratories, Bar Harbor, ME, USA), 16 P7 male and female C57BL/6 mice, 10 3–8 month-old male homozygous GFP mice [C57BL/6-Tg(CAG-EGFP)131Osb/LeySopJ; #006567, Jackson Laboratories], 12 3–8 month-old homozygous male Chx10-cre mice [Tg(Vsx2-cre)TC9Gsat/Mmucd, MMMRRC 36672], and 12 8–12 week-old homozygous female Ai14 mice [B6.Cg-*Gt(ROSA)26Sor^tm14(CAG-tdTomato)Hze^*/J, #007914, Jackson Laboratories]. Both male and female mice were used for the experiments in Figure 1, Figure 2 and Figure 3, whereas male mice only were used for the experiments in Figure 4. Animals were housed in ventilated cages under standard conditions (12-h light/dark cycle; lights on at 6:00 a.m.; 20–23 °C; 30–70% humidity) with unrestricted access to food and water. Four animals died intraoperatively or during recovery. An additional four animals were excluded after surgery due to inadequate graft survival.

### 2.3. Neural Progenitor Cell Isolation

*NPC isolation*. Timed pregnancies were used to generate mouse embryos as previously described [1,2,18,37]. Embryos were collected in the morning at embryonic day 12 following separation. Spinal cords were dissected free of meninges and divided longitudinally into anterior and posterior segments (~5 mm each), as previously described [38]. Tissue was enzymatically dissociated in 0.125% trypsin at 37 °C for 8–12 min, with a maximum of 10 cords processed per preparation. Digestion was terminated by the addition of 10 mL 10% fetal bovine serum in DMEM, followed by centrifugation at 600× *g* for 2 min. The pellet was gently triturated in Neurobasal medium supplemented with 2% B27 until a homogeneous suspension was obtained (~15–20 triturations). Cells were centrifuged again, resuspended in fresh NBM/B27, and filtered through a 40-μm mesh. Viability consistently exceeded 95% as assessed by trypan blue exclusion. Cells were maintained on ice until transplantation.

### 2.4. Spinal Cord Injury and Transplantation Surgeries

All procedures were performed under deep anesthesia induced with ketamine (25 mg/kg), xylazine (5.8 mg/kg), and acepromazine (0.25 mg/kg), with 0.5–1% isoflurane used to maintain anesthesia. We performed dorsal column wire knife lesions, an injury type in which grafts do not require a stabilizing matrix for survival [1,2,18,37,39,40]. A midline dorsal cervical incision was made following aseptic preparation of the surgical field. A laminectomy was performed at vertebral level C5 to expose the spinal cord. A tungsten wire knife (1.0–1.5 mm diameter; McHugh Milieux, Downers Grove, IL, USA) was positioned at the midline and lowered 0.8 mm beneath the dorsal surface prior to deployment. The elevation of the extruded wire transected the dorsal columns. Successful lesioning was confirmed by visual identification of a dorsal cavity beneath the dura.

*NPC transplantation*. Immediately prior to grafting, cells were resuspended at 5 × 10^5^ viable cells/μL in HBSS (Gibco, Waltham, MA, USA). Transplantation was performed within 5 min of injury to minimize additional procedures and because prior work has demonstrated robust graft survival under these conditions. A total of 2 μL (1.0 × 10^6^ cells) was delivered into the lesion cavity using a pulled glass micropipette attached to a PicoSpritzer II (Parker Hannifin, Hollis, NH, USA) over 5 min at a depth of 0.5–0.8 mm. Surgeries were distributed across multiple days, with donor cells obtained from 1–3 litters per day.

### 2.5. Cortical Injections

For anterograde labeling of corticospinal projections (Figure 3), AAV8-CAG-GFP (Addgene #37825, Watertown, MA, USA) was injected bilaterally into the sensorimotor cortex three weeks prior to endpoint. Bilateral cortical injections of virus were performed 3 weeks prior to sacrifice. Animals were anesthetized as described above and secured in a stereotaxic frame. Following midline scalp incision, bregma served as the reference point for coordinate determination. Small craniotomies were made over M1 and S1 regions. Virus (9.0 × 10^12^ gc/mL in sterile HBSS) was delivered using a Nanoject III (Drummond Scientific, Broomall, PA, USA) via pulled glass micropipettes. Twelve injection sites per hemisphere (250 nL each; total volume 3 μL) were administered at 0.7 mm depth at 3 nL/s, allowing at least 30 s between injections. Coordinates ranged from AP +0.5 to −0.3 mm and ML ±1.2 to 2.2 mm relative to bregma. Injection sites overlying the visible surface vasculature were avoided.

### 2.6. Pseudorabies Virus Injections

Pseudorabies virus (PRV-614, PRV-RFP; NIH Virus Center) was used for retrograde tracing of graft–host connectivity (Figure 4). Virus was diluted to 6 × 10^7^ gc/mL in sterile HBSS and injected into bilateral median nerves. Three injections (100 nL each) were delivered per nerve using a glass micropipette and PicoSpritzer II at approximately 1 μL/min.

### 2.7. Post-Operative Care

Muscle layers were closed with 4–0 silk sutures and skin was secured with stainless steel wound clips. Neo-Predef powder (Zoetis, Parsippany, NJ, USA) was applied prior to closure. Animals received daily subcutaneous injections of banamine (0.05 mg/kg) and ampicillin (0.05 mg/kg) in 0.5 mL lactated Ringer’s solution for three days. Cages were partially positioned on heating pads for 72 h post-operatively. Health checks were conducted daily for one week and weekly thereafter, with grooming and body weight monitored throughout the study.

### 2.8. Behavioral Assessments

*Pellet reaching*. We used a modified Whishaw skilled pellet reaching test to assess forelimb motor performance [41]. A semi-automated conveyer belt system designed by the Blackmore lab was used. Prior to the behavior session, the mice were placed in the behavioral testing room and given thirty minutes to acclimate. Before collecting the baseline data, the mice underwent a one-week acclimation period. On day 1 of testing, the mice were placed in the enclosure for twenty minutes to facilitate acclimatization (they remained in the same box for the entire study). On day 2, the mice were placed in the boxes again for twenty minutes, with 20 mg sugar pellets (Bio-Serv, Flemington, NJ, USA) placed inside the box to encourage them to eat. The same procedure was repeated on day 3, but this time the sugar pellet tray was positioned outside the cage to prompt the mice to reach for the pellets. On day 4, the sugar pellets were placed on an automated conveyor belt, and the mice were allowed to reach for each a single pellet during fifteen-second intervals, for a total period of 60 min. Video was recorded using OBS studio v. 32.0.4 (CapCut), with a total of eight cameras, four positioned on each row. During baseline recording, each pellet tray was filled using the alignment tool and aligned with the corresponding slit in the glass boxes. The mice were placed in their designated boxes and performed the task for a total of three rounds, with each round consisting of approximately 20 pellets. Prior to SCI, the mice were tested 5 days a week for 4 weeks on each task. After SCI, the mice were tested once a week on each task for the subsequent 6 weeks.

*Grip strength*. We utilized a grip strength measurement as a secondary forelimb motor behavioral outcome. Testing was done using the IITC Life Science Grip Strength Meter #2200 (Woodland Hills, CA, USA). Prior to the behavior session, the mice were placed in the behavior room and given thirty minutes to acclimate. To test their grip strength, the mice were held by the tail and allowed to grasp the t-bar. Once the mouse grasped the t-bar with both forepaws, the mouse was slowly pulled back at a horizontal angle keeping the mouse parallel to the ground. The maximum value was recorded from the grip-strength meter once the mouse released its forepaws. Each mouse was given three attempts, and the average grip strength was calculated. Grip strength was recorded daily for 3 days prior to SCI, then once a week for the following 6 weeks after.

### 2.9. Quantitative Real-Time PCR (qPCR)

Total RNA was isolated from 15–20 mg of flash-frozen spinal cord or graft tissue using RNeasy Plus Mini Kit (Qiagen, Germantown, MD, USA). GFP^+^ graft tissue was identified visually using handheld blue fluorescence flashlights (NightSea, Lexington, MA, USA). RNA yield and purity was analyzed by NanoDrop ND-1000 spectrophotometer (Nanodrop technologies, Wilmington, DE, USA), and first-strand cDNA was synthesized from 100 ng total RNA using SuperScript VILO MasterMix (Invitrogen, Carlsbad, CA, USA) following the manufacturer’s instructions. Quantitative real time PCR was executed using a Bio-Rad CFX Opus 96 Real-Time PCR System (Bio-Rad, Hercules, CA, USA). For each reaction the 20 μL reaction mixture contained 1 μL cDNA template, 8 μL molecular biology grade water, 1 μL primer mix (forward and reverse, 10 μM), and 10 μL 2× SYBR Green PCR Master Mix (Applied Biosystems #4364344, Waltham, MA, USA). RNA levels were normalized to the housekeeping gene *Gapdh* using the ΔCt method. ΔCt was calculated by subtracting the Ct value of *Gapdh* from that of the target gene (ΔCt = Ct(target) − Ct(*Gapdh*)), then converted to linear scale using the formula 2^(−ΔCt)^. PCR reactions were performed in triplicate for each cDNA sample. Primers were designed from nucleotide sequences obtained using NCBI BLAST (http://blast.ncbi.nlm.nih.gov/Blast.cgi, accessed on 1 April 2024) and synthesized by Integrated DNA Technologies (Coralville, IA, USA). The following primer pairs were used: GAPDH-F: CATCACTGCCACCCAGAAGACTG, GAPDH-R: ATGCCAGTGAGCTTCCCGTTCAG; HoxC6-F: AATTCCACCGCCTATGATCCA, HoxC6-R: ACATTCTCCTGTGGCGAATAAAA; HoxC10-F: ATGACATGCCCTCGCAATGTA, HoxC10-R: CCCCGCAGTTGAAGTCACTC. Sequences for the primers used in these experiments were obtained from PrimerBank [42,43].

### 2.10. Tissue Processing and Immunohistochemistry

*Tissue processing*. At study endpoint, animals were deeply anesthetized and perfused transcardially with 0.1 M phosphate buffer followed by 4% paraformaldehyde. Spinal columns were post-fixed overnight at 4 °C and cryoprotected in 30% sucrose until sectioning. Tissue was embedded in OCT compound and frozen on dry ice. Sagittal or coronal sections (20 μm) were collected into multi-well plates and stored at 4 °C.

*Immunohistochemistry*. Sections were processed as 1-in-6 or 1-in-12 series depending on analysis. After washes in TBS, sections were incubated for 1 h in blocking solution (5% donkey serum, 0.25% Triton X-100 in TBS). Primary antibodies (Supplementary Table S1) were applied overnight at 4 °C. Following TBS washes, appropriate Alexa Fluor-conjugated secondary antibodies (Jackson Immunoresearch, West Grove, PA, USA) were applied for 2 h at room temperature. Nuclei were counterstained with DAPI during the final wash. Sections were mounted on gelatin-coated slides and coverslipped with homemade Mowiol.

### 2.11. Image Acquisition

Fluorescent imaging was performed using a Nikon Eclipse upright microscope equipped with a motorized XY stage (Prior Scientific, Cambridge, UK) and Zyla 4.2 PLUS camera (Andor, Belfast, UK). Image capture and stitching were performed using NIS-Elements software. Acquisition parameters were held constant across samples processed together. For representative images, Z-stacks were occasionally collapsed using the Extended Depth of Focus function. Images were exported in TIFF format. Cultured cells were imaged using a Nikon Eclipse Ti2 inverted microscope.

### 2.12. Image Analysis

Quantitative analyses were conducted by two blinded investigators using ImageJ/FIJI. Graft regions were defined based on GFP or tdTomato fluorescence. Automated counting procedures were validated against manual counts. Samples lacking graft tissue or showing inadequate staining were excluded. Graft volume analysis: Extrapolated graft volume was determined by measuring the area within manually drawn regions of interest around grafts; every sixth section was analyzed for a given subject. The area of each section was multiplied by the section thickness, then multiplied by 6 to extrapolate the total graft volume (expressed as mm^3^).

NeuN, Sox9, Olig2, and Chx10 quantification: Immunoreactivity within graft ROIs (approximately four to six ROIs per animal depending on graft size) was thresholded for each marker using the ImageJ Auto Local Threshold function with Bernsen’s thresholding method [44]. Only cells that were double-positive for GFP^+^ or tdT^+^ (depending on whether the graft type was GFP^+^ or tdT^+^) were counted. For quantification of Chx10-^low^ and Chx10-^high^ cells, we used a flat threshold of Chx10 immunoreactivity. Watershed was applied to binary images and the Analyze Particles function was used to count the total number of cells.

Axon outgrowth quantification: GFP fluorescence was overexposed so that fine GFP^+^ processes were visible at sites distant from the main body of the graft. Graft ROIs were translated in 500-μm increments for 3.5 mm in rostral and caudal directions. At each increment, the total number of GFP^+^ axons crossing the leading edge of the ROI was manually counted. Data are represented as the total graft axon outgrowth, normalized to graft volume.

5-HT axon quantification: All sections containing grafts were analyzed within a 1-in-6 tissue series. Approximately four to six ROIs per animal were quantified, depending on graft size. Axons were manually traced in FIJI, and the total length of axons within the graft was normalized to graft volume.

CGRP and CST axon quantification: All sections containing grafts were analyzed within a 1-in-6 tissue series. Approximately four to six ROIs per animal were quantified, depending on graft size. Axons were thresholded in FIJI and the density of axons within the graft was normalized to graft volume.

### 2.13. Statistical Analysis

GraphPad Prism 10 was used to perform statistical analysis. Details for all statistical tests are provided in Supplementary Table S2. All data are presented as mean ± SEM. Statistical significance was defined as *p* < 0.05. All *t* tests were two-tailed.

## 3. Results

### 3.1. Region-Specific Hox Gene Expression in NPC Grafts

To generate region-specific NPC grafts, we isolated NPCs from the anterior or posterior halves of spinal cords from E12.5 GFP^+^ mouse embryos, derived from GFP^+^ transgenic mice, as previously described [38]. Either A-NPCs or P-NPCs were transplanted acutely into sites of cervical (C5) dorsal column wire knife lesion SCI in adult mice; this is the same lesion model used by our group previously [1,2,18,37]. Fourteen days later, grafts were collected by microdissection using GFP^+^ fluorescence to identify graft boundaries. For age-matched intact spinal cord tissue, we isolated either the anterior or posterior segments of P7 mouse spinal cords (Figure 1a). Given that the gestational length of mice is approximately 19–21 days, two-week-old E12.5 grafts correspond to approximately P7 in age. Tissue was subjected to qPCR to examine the expression of region-specific *Hox* genes. Specifically, we probed for *HoxC6*, which is expressed at forelimb spinal cord levels [45], and *HoxC10*, which is restricted to the lumbar spinal cord during development in vertebrates [46]. In P7 spinal cords, *HoxC6* expression was detectable in both regions but was significantly higher in anterior versus posterior tissue (Figure 1b; *p* = 0.0185), demonstrating that *HoxC6* is normally expressed broadly in the postnatal spinal cord with anterior enrichment. In contrast, *HoxC10* expression was readily detectable in most posterior P7 samples but absent from anterior samples, indicating expression that is normally restricted to the posterior cord (Figure 1c). We observed similar results in graft tissues; whereas *HoxC6* was expressed at comparable levels in A-NPC and P-NPC grafts (Figure 1d), *HoxC10* expression was significantly higher in P-NPC grafts versus A-NPC grafts (Figure 1e; *p* = 0.0500). Together, these results indicate that the axial identity of transplanted NPCs is at least partially retained following transplantation into the injured adult spinal cord.

### 3.2. Graft Axial Identity Does Not Affect Graft Neuronal or Glial Composition

We have previously demonstrated that restricting the dorsal/ventral identities of NPC grafts significantly influences the abundances of neuronal and glial cell populations in grafts [1,39]. This is because distinct populations of NPCs reside at distinct locations along the dorsoventral axis during development [47]. We next sought to determine whether there were differences in cell type abundances between anterior and posterior graft types. To test this, we transplanted A-NPCs or P-NPCs into sites of cervical SCI, then histologically examined graft tissue 4 weeks later (Figure 2a and Figure S1). Grafts of both types were successfully integrated into the host spinal cord, generating axon growth into surrounding tissue and cellular differentiation into neuronal (NeuN^+^), astrocytic (Sox9^+^), oligodendrocyte (Olig2^+^), and V2a interneuron (Chx10^+^) lineages. Quantification of graft volume revealed no differences between treatment groups (Figure 2b). To test for differences in the cellular composition of grafts we utilized immunohistochemistry to probe for the presence of neurons, astrocytes, oligodendrocytes, and V2a interneurons. There were no significant differences in graft volume neuronal density (Figure 2c,d), astroglial density (Figure 2e,f), oligodendrocyte density (Figure 2g,h), or V2a interneuronal density (Figure 2i,j) between anterior and posterior graft types (statistical analyses are described in the figure legends, and in detail in Supplementary Table S2). To test for potential differences in total axon outgrowth between the two graft types, we measured the numbers of GFP^+^ axons present in sagittal spinal cord sections at 500-µm intervals from the graft/host border. We did not observe any differences in graft-derived axon outgrowth into the host spinal cord (Figure 2k,l). Hence, these two graft types exhibit similar abundances of major cell types and ability to extend axons.

### 3.3. Graft Axial Identity Does Not Influence Host Axon Regeneration into Grafts

We have previously shown that host axons spontaneously regenerate into NPC graft tissue within days to weeks post-transplantation [1,2,26,39,48]. We therefore histologically evaluated the regeneration of host axon populations into grafts to determine whether graft axial identity influences this process. In four-week-old GFP^+^ grafts, we quantified ingrowth from CGRP^+^ nociceptive fibers and from 5-HT^+^ brainstem-derived axons. Based on an extensive body of previous work characterizing graft neuronal phenotypes in vitro and in vivo, any 5-HT^+^ or CGRP^+^ axons within grafts are by necessity host-derived and not graft-derived [1,18,37,39]. We did not observe any differences in axon regeneration into A-NPC versus P-NPC grafts (Figure 3a–d). We next performed an experiment to examine corticospinal tract (CST) axon regeneration into grafts, as the corticospinal system is considered a therapeutically important population in SCI. We transplanted into sites of SCI either A-NPCs or P-NPCs derived from Chx10-cre::Ai14 embryonic spinal cords, which produced grafts with tdTomato^+^ V2a neurons; four weeks later, we anterogradely labeled CST axons with AAV-GFP (Figure 3e). Using this system, cre-dependent gene expression is restricted to V2a graft-derived neurons, as previously published [18]. We utilized tdT^+^ V2a grafts in this experiment because V2a neurons are synaptic targets of corticospinal axons [49], and because there is normally graded expression of the Chx10 protein in postmitotic V2a neurons along the rostrocaudal axis [50]. Even though we previously observed that the total abundance of Chx10-immunoreactive V2a neurons does not differ between grafts (Figure 2i,j), we did not account for the proportions of Chx10-high versus Chx10-low tdT^+^ labeling, which this system allows us to quantify.

Figure 3f,g. However, we did detect a significant increase in the proportion of Chx10-low graft V2a neurons in anterior grafts versus posterior grafts (*p* = 0.0196, Figure 3h,i). There was an insignificant trend toward increased Chx10-high neurons in posterior grafts (Figure 3j). This finding is consistent with the previous observation that rostral V2a neurons express lower levels of Chx10 than caudal levels in the intact spinal cord [50]. Interestingly, corticospinal fibers were found to be closely associated with graft V2a neurons in some instances (Figure 3k,l), suggesting that these cells may represent postsynaptic targets for CST axons in grafts. We also note that we did not quantify tdTomato^+^ axon extension from grafts in this study, as there were very few axons extending beyond the host/graft border in all animals.

### 3.4. Graft Axial Identity Does Not Affect Recovery of Forelimb Motor Function After SCI

We performed a long-term behavioral study to determine whether graft axial identity influences the recovery of motor function after spinal cord injury. Following cervical dorsal column lesion SCI, rodents exhibit impaired performance on forelimb motor tasks including skilled pellet reaching and grip strength [26,51,52,53,54], so we chose to focus on these behavioral tasks in this study. For this study, we utilized all male mice. For the forelimb reach task, we utilized a modified Whishaw test in which mice were placed in a Plexiglas enclosure with a narrow window through which they were allowed to reach for small treat pellets. Every 15 s, a conveyer belt would advance and present a new pellet, allowing mice to attempt to reach and grasp for a total of 60 min per session. For the grip strength task, we analyzed the bilateral grip strength of mice using a standard grip strength meter.

Following acclimation and baseline behavioral testing on these assays, we transplanted GFP^+^ A-NPCs or P-NPCs into sites of C5 SCI and continued behavioral testing once weekly for 6 weeks (Figure 4a). We did not include an injury-only, ungrafted group in this study because the purpose of this experiment was simply to compare performance in animals receiving A-NPC versus P-NPC grafts. Animals then received bilateral injections of the retrograde transsynaptic tracer, pseudorabies virus (PRV) expressing red fluorescent protein, into forelimb median nerves and were sacrificed for histological analysis 96 h later. We first analyzed the percentage of reach attempts that successfully resulted in retrieval of a treat pellet. Data were normalized to percent success on the final day of training prior to injury. Although SCI produced significant deficits in performance on this task, we did not observe any significant differences in recovery between A-NPC and P-NPC groups (Figure 4b). However, it is important to note that there was a large amount of inter-subject variability (Figure 4b). On the grip strength task, animals exhibited baseline grip strengths of 91.2 ± 3.82 g (A-NPC) and 99.3 ± 3.58 g (P-NPC) prior to injury (Figure 4c). At 3 days post-SCI, averages dropped to 14.9 ± 1.64 g (A-NPC) and 15.9 ± 1.94 g (P-NPC) and gradually recovered to 51.5 ± 5.20 g (A-NPC) and 51.4 ± 4.23 g (P-NPC) by 45 days post-SCI. However, there was no statistically significant difference in the effect of treatment over time in grip strength between groups.

In order to determine whether there existed a differential degree of graft/host synaptic integration between groups, we analyzed graft PRV infection as previously described [18]. Following PRV injection into the median nerves, we quantified PRV^+^ neurons in grafts and normalized these values to primary-infected PRV^+^ motor neurons in the host spinal cord. Both graft types contained PRV^+^ neurons in similar densities to what we have previously reported [18] (Figure 4d). However, there were no significant differences in the density of PRV^+^ neurons in grafts between treatment groups (Figure 4e). Anecdotally, we did observe that the majority of PRV^+^ neurons were near the graft/host border. Finally, upon correlating graft PRV infection with endpoint behavioral scores of individual animals, we did not detect any significant relationship between these data (Figure 4f,g). Together, these findings indicate that graft axial identity does not significantly impact the synaptic integration into the host cervical spinal cord or promote different behavioral outcomes in mice following SCI.

## 4. Discussion

As the field moves forward with human clinical application of stem and progenitor cell therapies for spinal cord injury, identifying the experimental and biological factors that dictate graft success is critical. Previous research has established that regional identity (e.g., brain vs. spinal cord) and dorsoventral identity are critical, as they dictate the cellular composition of the graft and degree of synaptic integration between graft and host [10,20,21,22,23,24,25,26]. Given that different axial levels of the spinal cord possess distinct circuitries, neuronal identities, and functional outputs, we hypothesized that matching the axial identity of the graft to the injury site would optimize integration and recovery. However, our results surprisingly demonstrate that graft axial identity is not a primary determinant of anatomical or functional success in a cervical SCI model.

Our findings confirm that transplanted NPCs retain their intrinsic axial markers after engraftment into the adult environment. Specifically, P-NPC grafts maintained significantly higher expression of the lumbar-associated gene *HoxC10* compared to A-NPC grafts. While *HoxC6* was expressed in both graft types, this aligns with our postnatal P7 control data showing *HoxC6* expression is broader than its embryonic restriction to cervical segments. Furthermore, the increased proportion of Chx10-high V2a neurons in posterior grafts mirrors the known caudal-to-rostral gradient of Chx10 protein expression in the intact cord [50]. Despite these maintained molecular differences, the two graft types were indistinguishable in terms of cell type composition, host-graft axon outgrowth, and host axon regeneration.

The lack of differential recovery in pellet reaching and grip strength suggests that the “rules” of embryonic development, where segment-specific identity governs precise connectivity, may not strictly apply to the adult injured environment. However, it is important to note that we observed a high degree of variability in behavioral recovery among individual animals in this study, which may mask any effects of treatment. There may be sufficient plasticity in how grafted NPCs differentiate or form circuits to overcome axial mismatches. Interestingly, we observed corticospinal fibers in close proximity to graft V2a neurons regardless of graft identity. Since V2a interneurons are vital targets for restoring function after SCI [7,55,56], their presence and potential as synaptic relays may be more important than their specific axial origin.

A primary limitation of this study is the broadness of our cell populations: “anterior” (cervical to upper thoracic) and “posterior” (lower thoracic to sacral). This was necessitated by the technical difficulty of isolating specific spinal segments from E12.5 embryos. While our qPCR likely included some host-derived mRNA due to graft vascularization, the clear *HoxC10* disparity confirms that the grafts remained distinct. As molecular strategies for directed differentiation of human iPSCs improve, it remains to be seen if “level-matched” grafts, such as specific C5-identity NPCs, would offer a subtle advantage not captured in this study. Future work should focus on characterizing the specific neuronal populations in grafts that receive inputs from key motor pathways, such as the reticulospinal or corticospinal tracts, to determine if their downstream connections to host motor pools are influenced by axial markers.

Although this study was not designed to model clinical efficacy per se, it is important to contextualize these findings within the broader framework of NPC therapy for SCI. The prevailing hypothesis underlying NPC transplantation is that graft-derived neurons differentiate into regionally appropriate interneuronal subtypes, integrate synaptically with host axons, and form functional relay circuits that bridge interrupted descending and ascending pathways. In this model, regenerating host axons grow into graft tissue and form synapses onto graft-derived neurons, and graft axons extend beyond the lesion to reconnect with distal host circuitry. Such relay formation has been demonstrated anatomically and functionally in multiple experimental models [4,7,18,39,57,58,59]. Our data suggest that axial segment matching may not be required for this relay function to occur in a cervical dorsal column injury model. Both anterior- and posterior-derived NPCs generated neurons capable of supporting host axon regeneration, including corticospinal tract ingrowth, and exhibited similar degrees of retrograde transsynaptic connectivity. These findings suggest that the capacity to assume spinal interneuron identities and participate in local circuit formation may be more important than precise rostrocaudal matching.

## Data Availability

The original contributions presented in this study are included in the article/Supplementary Materials. Further inquiries can be directed to the corresponding author.

## Supplementary Materials

The following supporting information can be downloaded at: https://www.mdpi.com/article/10.3390/cells15060497/s1, Figure S1: Representative images of sagittal spinal cords containing A-NPC grafts (top row) or P-NPC grafts (bottom row). Each panel is from a different subject. Scale bars = 500 μm; Table S1: Antibodies and primers used in this study; Table S2: Detailed description of statistical analyses used in this study.
