# Supplementary material for: Axial Identity of Spinal Cord Neural Progenitor Cell Grafts Is Dispensable for Regeneration and Functional Recovery After Spinal Cord Injury
Source: Cells. 2026 Mar 11;15(6):497. doi: 10.3390/cells15060497 (PMC13024784; doi:10.3390/cells15060497)
Supplement: Supplementary file 1 [file cells-15-00497-s001.zip › cells-4153875-supplementary.pdf]

Supplementary Information

Axial identity of spinal cord neural progenitor cell grafts is dispensable for regeneration and functional recovery after spinal cord injury

Ashley Smith*, Valerie Dietz*, Joseph Hoppe, Gillian Imrie, Grant Lee, Amy Leonards, Vipin Jagrit, Abigail Evans, Tucker Gillespie, Bryson Gottschall, Benard Inskeep, Prakruthi Amar Kumar, Logan Friedrich, Murray Blackmore, Isabella Farhy-Tselnicker, Jennifer N. Dulin

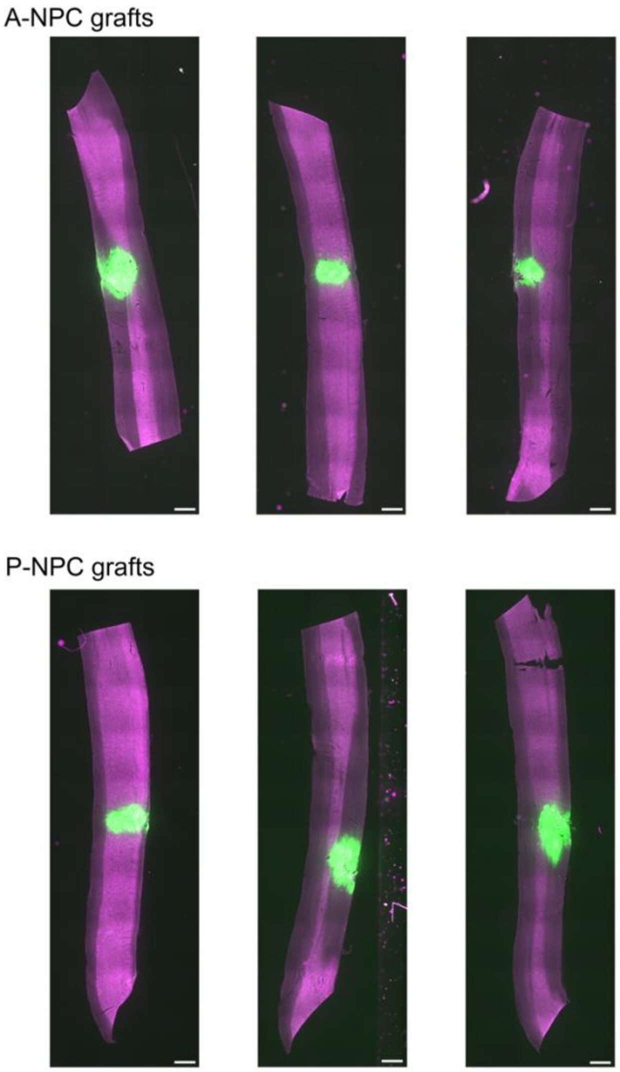

Supplementary Figure S1. Representative images of sagittal spinal cords containing A-NPC grafts (top row) or P-NPC grafts (bottom row). Each panel is from a different subject. Scale bars = 500 μm.

Supplementary Table S1. Antibodies and primers used in this study.

Antibody	Catalog #	RRID	Dilution
**Primary antibodies**			
Rabbit anti-5-HT	Immunostar #20080	AB_572263	1:20,000
Sheep anti-CGRP	Abcam #ab195387	AB_2915992	1:1000
Sheep anti-Chx10	Abcam #ab16141	AB_302278	1:1500
Chicken anti-GFP	Aves #GFP-1020	AB_10000240	1:1000
Goat anti-mCherry	Sicgen #AB0040	AB_2333093	1:1000
Guinea pig anti-NeuN	Millipore #ABN90	AB_11205592	1:1000
Chicken anti-Olig2	Aves #OLIG2-0100	AB_2924438	1:1000
Rabbit anti-RFP	Abcam #ab62341	AB_945213	1:1000
Goat anti-Sox9	Biotechne #AF3075	AB_2194160	1:400
**Secondary antibodies**			
Donkey anti-chicken 488	Jackson Immunoresearch #703-545-155	AB_2340375	1:1000
Donkey anti-chicken 647	Jackson Immunoresearch #703-605-155	AB_2340379	1:1000
Donkey anti-goat 555	Jackson Immunoresearch #705-565-147	AB_3095465	1:1000
Donkey anti-guinea pig 488	Jackson Immunoresearch #706-545-148	AB_2340472	1:1000
Donkey anti-guinea pig 647	Jackson Immunoresearch #706-605-148	AB_2340476	1:1000
Donkey anti-rabbit 555	Jackson Immunoresearch #711-565-152	AB_3095471	1:1000
Donkey anti-rabbit 647	Jackson Immunoresearch #711-605-152	AB_2492288	1:1000
Donkey anti-sheep 555	Jackson Immunoresearch #713-565-147	AB_3095477	1:1000
Donkey anti-sheep 647	Jackson Immunoresearch #713-605-147	AB_2340751	1:1000
**Primers**	**Sequence**		
GAPDH-F	CATCACTGCCACCCAGAAGACTG		
GAPDH-R	ATGCCAGTGAGCTTCCCGTTCAG		
HoxC6-F	AATTCCACCGCCTATGATCCA		
HoxC6-R	ACATTCTCCTGTGGCGAATAAAA		
HoxC10-F	ATGACATGCCCTCGCAATGTA		
HoxC10-R	CCCCGCAGTTGAAGTCACTC		

Supplementary Table S2. Detailed description of statistical analyses used in this study.

Fig.	Parameter	Group size (n)	Statistical test	Significance level
1b	HoxC6 expression (P7 cords)	A (n=8); P (n=8)	Unpaired, two-tailed Welch's t test	t=2.673, df=13.71<br>p=0.0185
1c	HoxC10 expression (P7 cords)	A (n=6); P (n=8)	Unpaired, two-tailed Welch's t test	t=4.009, df=7.246<br>p=0.0048
1d	HoxC6 expression (G14 grafts)	A (n=12); P (n=7)	Unpaired, two-tailed Welch's t test	t=0.1381, df=14.22<br>p=0.8921
1e	HoxC10 expression (G14 grafts)	A (n=12); P (n=8)	Unpaired, two-tailed Welch's t test	t=2.364, df=7.012<br>p=0.0500
2b	Graft volume	A (n=10); P (n=8)	Unpaired, two-tailed Student's t test	t=0.9762, df=16<br>p=0.3435

2d	Graft neuronal density	A (n=10); P (n=8)	Unpaired, two-tailed Student's t test	t=0.0276, df=16<br>p=0.9784
2f	Graft astrocyte density	A (n=10); P (n=8)	Unpaired, two-tailed Student's t test	t=0.4334, df=16<br>p=0.6705
2h	Graft oligodendrocyte density	A (n=9); P (n=8)	Unpaired, two-tailed Student's t test	t=0.4893, df=15<br>p=0.6317
2j	Graft V2a neuronal density	A (n=9); P (n=8)	Unpaired, two-tailed Student's t test	t=0.6193, df=15<br>p=0.5450
2l	Graft axon outgrowth	A (n=10); P (n=8)	Two-way ANOVA with Sidak's multiple comparisons test	Distance x treatment main effect: F (2.375, 38.01) = 0.7989, p=0.4765<br>Distance main effect: F (2.375, 38.01) = 44.91, p<0.0001<br>Treatment main effect: F (1, 16) = 1.118, p=0.3061<br>Subject main effect: F (16, 208) = 4.242, p<0.0001
3b	CGRP+ axon density in grafts	A (n=9); P (n=8)	Unpaired, two-tailed Student's t test	t=0.5100, df=15<br>p=0.6175
3d	5-HT+ axon length in grafts	A (n=9); P (n=9)	Unpaired, two-tailed Student's t test	t=0.2987, df=16<br>p=0.7690
3g	CST axon density in grafts	A (n=11); P (n=6)	Unpaired, two-tailed Student's t test	t=0.7959, df=15<br>p=0.4385
3i	Chx10-low cells in graft	A (n=11); P (n=6)	Unpaired, two-tailed Welch's t test	t=2.655, df=13.24<br>p=0.0196
3j	Chx10-high cells in graft	A (n=11); P (n=6)	Unpaired, two-tailed Welch's t test	t=1.012, df=7.069<br>p=0.3450
4b	Pellet reaching success	A (n=8); P (n=12)	Two-way repeated measures ANOVA with Sidak's multiple comparisons test	Time x treatment main effect: F (7, 126) = 1.152, p=0.3355<br>Time main effect: F (7, 126) = 31.42, p<0.0001<br>Treatment main effect: F (1, 18) = 1.070, p=0.3147<br>Subject main effect: F (18, 126) = 7.695, p<0.0001
4c	Grip strength	A (n=8); P (n=12)	Two-way repeated measures ANOVA with Sidak's multiple comparisons test	Time x treatment main effect: F (4.259, 76.67) = 1.461, p=0.2197<br>Time main effect: F (4.259, 76.67) = 128.5, p<0.0001<br>Treatment main effect: F (1, 18) = 0.0506, p=0.8246<br>Subject main effect: F (18, 162) = 3.528, p<0.0001
4e	Graft PRV infection	A (n=8); P (n=12)	Unpaired, two-tailed Student's t test	t=0.0817, df=18<br>p=0.9358
4f	Graft PRV infection versus day 42 grip strength scores	A (n=8); P (n=12)	Simple linear regression analysis	R^2=0.1196<br>p=0.1354
4g	Graft PRV infection versus day 42 pellet reach cores	A (n=8); P (n=12)	Simple linear regression analysis	R^2=0.01045<br>p=0.6680
